# Adiponectin inhibits TGF-β1-induced skin fibroblast proliferation and phenotype transformation via the p38 MAPK signaling pathway

**DOI:** 10.1515/biol-2022-0679

**Published:** 2023-08-10

**Authors:** Xueling Wang, Xiaoting Yan, Fang Huang, Lijuan Wu

**Affiliations:** School of Medicine, Taizhou University, No. 1139, Shifu Avenue, Taizhou, Zhejiang 318000, China; Taizhou Central Hospital, Taizhou, 318000, China

**Keywords:** adiponectin, dermal fibroblasts, fibrosis, pathological scars, P38 MAPK

## Abstract

The aim of this study was to investigate the effects of adiponectin (APN) on the proliferation and phenotypic transformation of human skin fibroblasts (HSFs) induced by TGF-β1. Primary fibroblast cultures were collected from prepuce surgery, and the cell viability and proliferative activity of HSFs were detected by Cell Counting Kit-8 and EdU assays. In addition, cell migration was detected by Transwell assay. The protein levels of related genes in HSF were detected by Western blotting. The results showed that the proliferation and migration abilities of HSF in the TGF-β1 group were significantly improved, and the relative protein expression levels of PCNA, α-SMA, and Collagen I in the TGF-β1 group were greatly increased. Furthermore, TGF-β1 stimulated the phosphorylation of p38 in HSF, while APN pretreatment significantly inhibited the TGF-β1-induced phosphorylation of p38. Additionally, blocking the p38 MAPK signaling pathway relieved the injury in the HSF induced by TGF-β1 and enhanced the therapeutic effect of APN in the TGF-β1-treated HSF. In conclusion, APN inhibits TGF-β1-induced HSF proliferation and myofibroblast phenotypic transformation by activating the p38 MAPK signaling pathway. APN is expected to become a potential target for preventing and treating skin fibrosis and pathological scars.

## Graphical abstract

**Figure:**
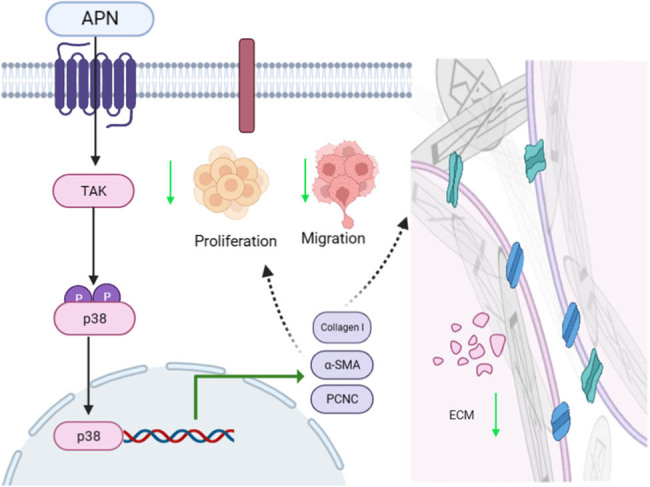

## 1 Introduction

One of the most frequent side effects following surgery, trauma, or skin burn is pathological scarring, which develops as a result of excessive tissue healing after damage. Hypertrophic scars and keloids are the two most common types of pathological scars, which are dermal fibroproliferative conditions. The typical pathological features are abnormal proliferation of fibroblasts (Fbs) and their transformation to myofibroblasts (MFbs) and excessive deposition of extracellular matrix (ECM) dominated by collagen [1]. Itching, pain, dysfunction caused by scar contracture, and even malignant transformation caused by recurrent ulceration can accompany pathological scars. These side effects greatly affect the quality of life of patients and result in a significant financial and psychological burden [2]. Currently, many explanations exist for the pathogenesis of pathological scars. Several factors, such as cells, growth factors, microcirculation, immunity, and gene regulation, are closely related to the pathogenesis of pathological scars; however, the specific pathogenesis is still unclear.

Existing studies have shown that overactivation of MFbs and over deposition of collagen are the main mechanisms of pathological scar formation [3,4]. The process of wound healing is divided into inflammatory, proliferative, and remodeling phases, which are complex and interlaced with each other. Many proteins and cytokines play their own or synergistic roles in the process of wound healing. In the proliferative stage, Fbs activate into MFbs, which further constrict the wound and release collagen to restore ECM balance. Under normal steady-state conditions, the scope and intensity of wound repair are limited. Pathological fibrosis occurs when the crucial healing process is out of balance or worsened, leading to an excessive buildup of the ECM, which may lead to the formation of pathological scars [5]. Additionally, in Fbs, TGF-β1 is an effective method to stimulate ECM protein synthesis, including collagen, fibronectin, metalloproteinase-1, connective tissue growth factor, etc. [6]. The role of TGF-β1 in the pathogenesis of skin fibrosis has attracted widespread attention. Therefore, investigating new factors regulating the proliferation and phenotypic transformation of skin Fbs and using such factors as new targets for preventing and treating pathological scars will provide new ideas for the study of scar pathogenesis and treatment.

Adiponectin (APN) is an endogenous bioactive protein secreted by adipocytes. After binding with its receptor, APN plays a vital role in regulating metabolism, regulating immune balance, inhibiting cell proliferation and the inflammatory response, and maintaining vascular homeostasis by activating downstream signaling pathways [7,8]. Recent studies have found that APN has potential antifibrotic effects on lung, liver, kidney, and heart fibrosis-related diseases [9,10,11,12]. Luo et al. suggested that the expression of APN and its receptor adipoRs in keloid tissues and cells is lower than that in normal skin tissues. APN may inhibit the proliferation, migration, and ECM accumulation of keloid Fbs induced by CTGF by activating signal pathways such as p38 MAPK, suggesting that APN plays a crucial role in the pathogenesis of keloids [13]. Follow-up studies confirmed that APN might exert anti-inflammatory and antifibrotic roles in the development of keloids [14,15]. Although these studies have confirmed that APN can inhibit keloid formation, there are few reports on the antifibrotic effect of APN on skin tissue. Recent animal experiments have shown that knocking out the APN gene is prone to fibrosis, indicating that APN can inhibit the development of tissue and organ fibrosis [16]. Therefore, further research is required to fully understand how APN affects Fb proliferation and MFb phenotypic transition, which is a crucial step in the etiology of pathological scars.

Therefore, this study aimed to explore the effect of APN on TGF-β1-induced human skin fibroblast (HSF) proliferation and MFb phenotype transformation and whether it is regulated by the p38 MAPK signaling pathway to provide a theoretical basis for the molecular mechanism of skin fibrosis and pathological scar formation.

## 2 Materials and methods

### 2.1 Reagents and equipment

Human adiponectin (Acrp30) and human TGF-β1 were purchased from Novoprotein (Beijing, China). Cell Counting Kit-8 (CCK-8) and EdU kits were procured from SolarBio (Beijing Solebo Technology Co., Ltd, Beijing, China). PCNA antibody, smooth muscle actin polyclonal (α-SMA) antibody, collagen type I (collagen I) rabbit polyclonal antibody, p38 MAPK antibody, p-p38 MAPK antibody, NF-κB antibody, p-NF-κB antibody, β-catenin antibody, p-β-catenin antibody, Notch1 antibody, and HRP-conjugated AffiniPure goat anti-mouse IgG (H + L) were obtained from Proteintech (Wuhan, China). SB203580 was purchased from MCE (Shanghai, China). Other chemicals used in this study, such as the BCA protein assay kit and SDS-PAGE gel separation kit, were acquired from ASPEN (ASPEN Biotechnology Co., Ltd, Wuhan, China).

### 2.2 HSF culture and treatment

Four human foreskin samples were collected from the Department of Pediatrics of Taizhou Central Hospital (Taizhou, China). All patients provided informed consent. This research was approved by the Ethics Committee of Taizhou University (Taizhou, China). All procedures for HSF purification and culture were performed as described by Guo et al. [17]. Fat was removed from the tissue, and the remaining tissue was cut into 3 mm strips, which were incubated in 0.05% dispase and Dulbecco’s modified Eagle’s medium (DMEM) at 4°C overnight. The epidermis was excised, and the dermis was chopped and placed in a 25 cm flask pretreated with 10% fetal bovine serum (FBS) horizontally for 1 h, followed by vertical incubation in a 37°C, 5% CO_2_ chamber for 3 h. Tissues were cultured in DMEM containing 5.5 mM glucose, 10% FBS, and 1% penicillin‒streptomycin (Gibco), and then the medium was replaced every 3 days. When the cell confluency reached 70–80%, the cells were digested with 0.25% trypsin and passaged. Cells from passages 4–5 were used. Then, the HSFs were treated with 10 ng/mL TGF-β1 for 48 h to establish the skin fibrosis model. For APN treatment, HSF was treated with APN (1, 5, and 10 μg/mL) for 48 h.


**Informed consent:** Informed consent has been obtained from all individuals included in this study.
**Ethical approval:** The research related to human use has been complied with all the relevant national regulations, institutional policies, and in accordance with the tenets of the Helsinki Declaration, and has been approved by Ethics Committee of School of Medicine, Taizhou University, (Taizhou, China).

### 2.3 CCK-8 assay

Cell viability was determined with a CCK-8. After digestion with trypsin, 100 μL of the cell suspension (1 × 10^3^ cells/well) was transferred to 96-well cell culture plates, with four replicate wells in each group and blank wells. After attachment to the culture plate, the culture plates were incubated at 37°C and 5% CO_2_ overnight. As a blank control cell, no reagent was added; instead, 10 μL of CCK-8 solution and cell culture media were added to each well. After an additional 0.5 h of incubation in the cell incubator, the density was determined by measuring the absorbance (OD value) per well at 450 nm with a microplate reader. The best APN action time and concentration were chosen. Each experiment was repeated three times.

### 2.4 EdU assay

According to the instructions of the EdU kit (RiboBio Biotechnology Co., Ltd, Guangzhou, China), the cells were inoculated in 96-well plates (2 × 10^3^ cells/well) for 24 h and incubated with diluted EdU solution for 2 h. After that, the cells were fixed with paraformaldehyde (4%) for 30 min at room temperature. Then, the cells were treated with Apollo for 30 min. A 1× Hoechst 33342 reaction solution was used to label nuclei. Finally, the cells were observed under a fluorescence microscope. Five visual fields were randomly selected from each group. The images of cells stained differently in the same visual field were superimposed with ImageJ software to calculate the number of two kinds of stained cells. Cell proliferation capacity = number of green fluorescent cells/number of blue fluorescent cells × 100％.

### 2.5 Transwell assay

Cell migration was analyzed by Transwell assay. Transwell chambers (24-well, 8 μm pore) were purchased from Corning (Corning, USA). The treated cells were suspended in culture medium without FBS and added to the top chambers. Complete medium was added to the lower chambers. After 24 h, the cells in the top chambers were removed. The migrated cells were immobilized with 4% PFA (Sigma-Aldrich) and stained with 0.2% crystal violet (Sigma-Aldrich). The results were visualized and counted under a microscope (KEYENCE, Osaka, Japan).

### 2.6 Western blot assay

Western blotting was performed to detect the protein expression levels of PCNA, α-SMA, collagen I, p38 MAPK, p-p38 MAPK, NF-κB, p-NF-κB, β-catenin, p-β-catenin, and Notch1. The total protein in the cells was extracted after stimulation with the ideal APN concentration and duration, and the protein concentration of the sample was determined by the BCA protein assay kit. After protein denaturation, the proteins were electrophoretically separated and then transferred to a PVDF membrane. The turned film was added to the blocking solution (5% skimmed milk) and sealed at room temperature for 1 h. Five percent skimmed milk was removed, and the membrane was incubated with the following primary antibodies: PCNA, α-SMA, collagen I, p38 MAPK, p-p38 MAPK, NF-κB, p-NF-κB, β-catenin, p-β-catenin, and Notch1 antibodies at 4°C overnight, followed by washing with TBST buffer (tris-buffered NaCl solution with Tween-20) three times (10 min per wash). Consequently, the membrane was incubated with HRP-conjugated Affinipure goat anti-mouse IgG (H + L) as the secondary antibody at room temperature for 60 min and then rinsed with TBST at room temperature four times (10 min per wash). The membrane was incubated with freshly prepared ECL mixed solution (A:B = 1:1) and exposed in a dark room. The gray value of the target band was assayed by ImageJ software. The relative expression level of the target protein was determined by the gray value ratio of the target protein to β-actin (loading control). Each experiment was performed three times.

### 2.7 Statistical analysis

Data were analyzed using GraphPad Prism 9.0.2. The data are expressed as the mean value ± standard deviation (SD). Each experiment is conducted independently three times. The difference between groups was assessed using one-way analysis of variance, and the Holm-Sidak test was performed as a post hoc test. Differences between two groups were analyzed using a t test. Values with *p* < 0.05 were considered statistically significant. All experiments were repeated at least three times.

## 3 Results

### 3.1 APN Inhibits the cell viability of HSF induced by TGF-β1

**Figure 1:**
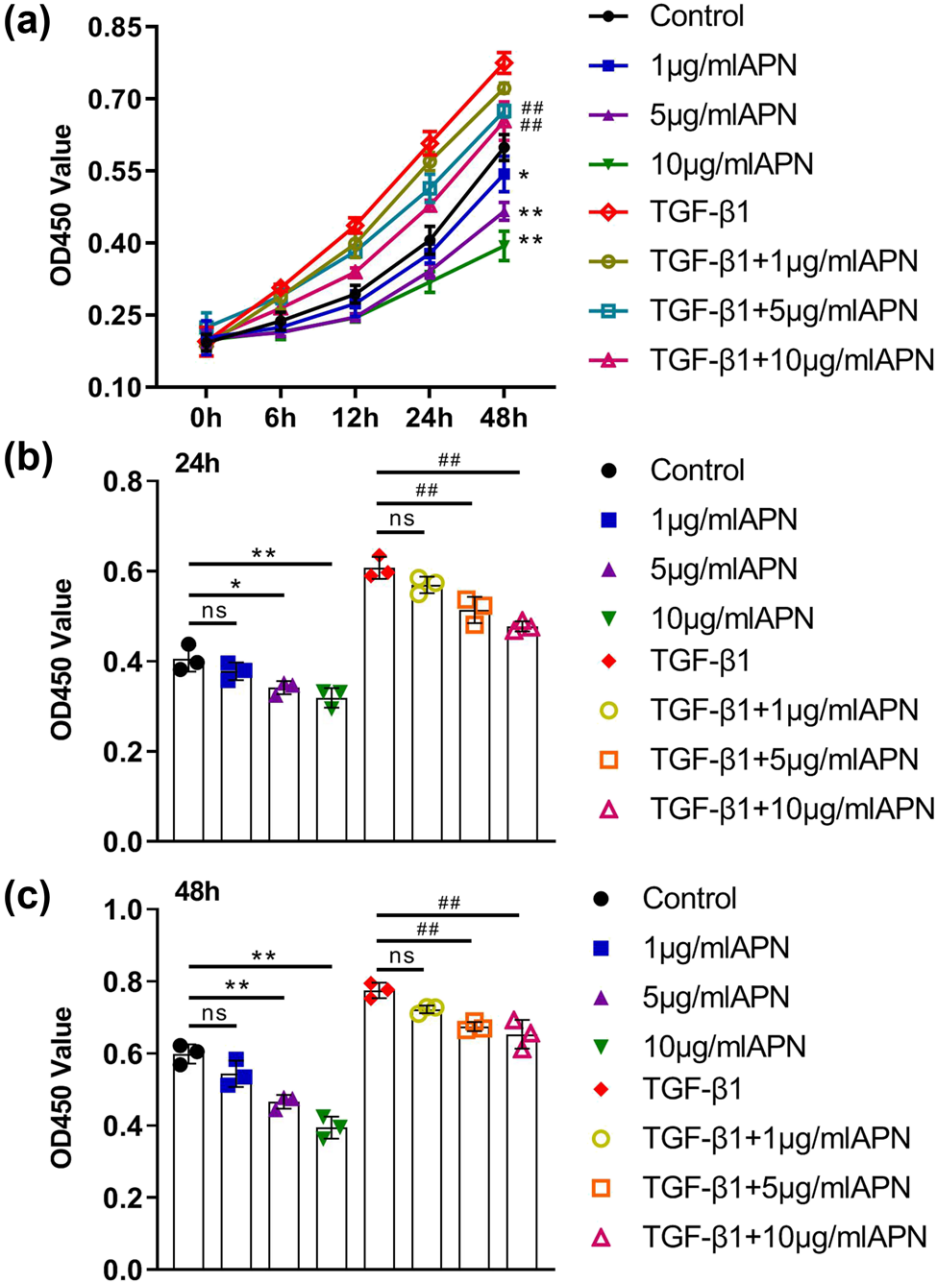
APN inhibits the cell viability of HSF induced by TGF-β1. (a) The viability of HSF cells treated withTGF-β1 (10 ng/mL) and APN (1, 5, 10 μg/mL) was detected by CCK-8 assay at 6 h, 12 h, 24 h and 48 h, which was expressed as a line chart. The viability of HSF cells treated with TGF-β1 (10 ng/mL) and APN (1,5,10 μg/mL) was detected by CCK-8 assay at 24 h (b) and 48 h (c) and expressed as bar chart. Values are presented as the mean value ± SD (*n* = 3). *p < 0.05, **p < 0.01 compared to the control group. ##*p* < 0.01 compared to the TGF-β1 group.

To analyze the inhibition of HSF proliferation by APN, a CCK-8 assay was performed. Compared with the control group, 10 ng/mL TGF-β1 treatment significantly enhanced the proliferation of HSF cells. Then, both HSF cells and TGF-β1-treated HSF cells were treated with different doses of APN, and the results showed that APN treatment significantly decreased the viability of the cells in a dose-dependent manner (Figure 1a). In addition, the viability of HSF cells treated with 10 ng/mL TGF-β1 and different doses of APN at 24 h (Figure 1b) and 48 h (Figure 1c) were quantified, which further showed that APN treatment significantly decreased the viability of both HSF cells and TGF-β1-treated HSF cells. Based on the above data, the ideal time and concentration of APN were established to be 48 h and 10 μg/mL, respectively.

### 3.2 APN inhibits the phenotypic transformation of HSF induced by TGF-β1

**Figure 2:**
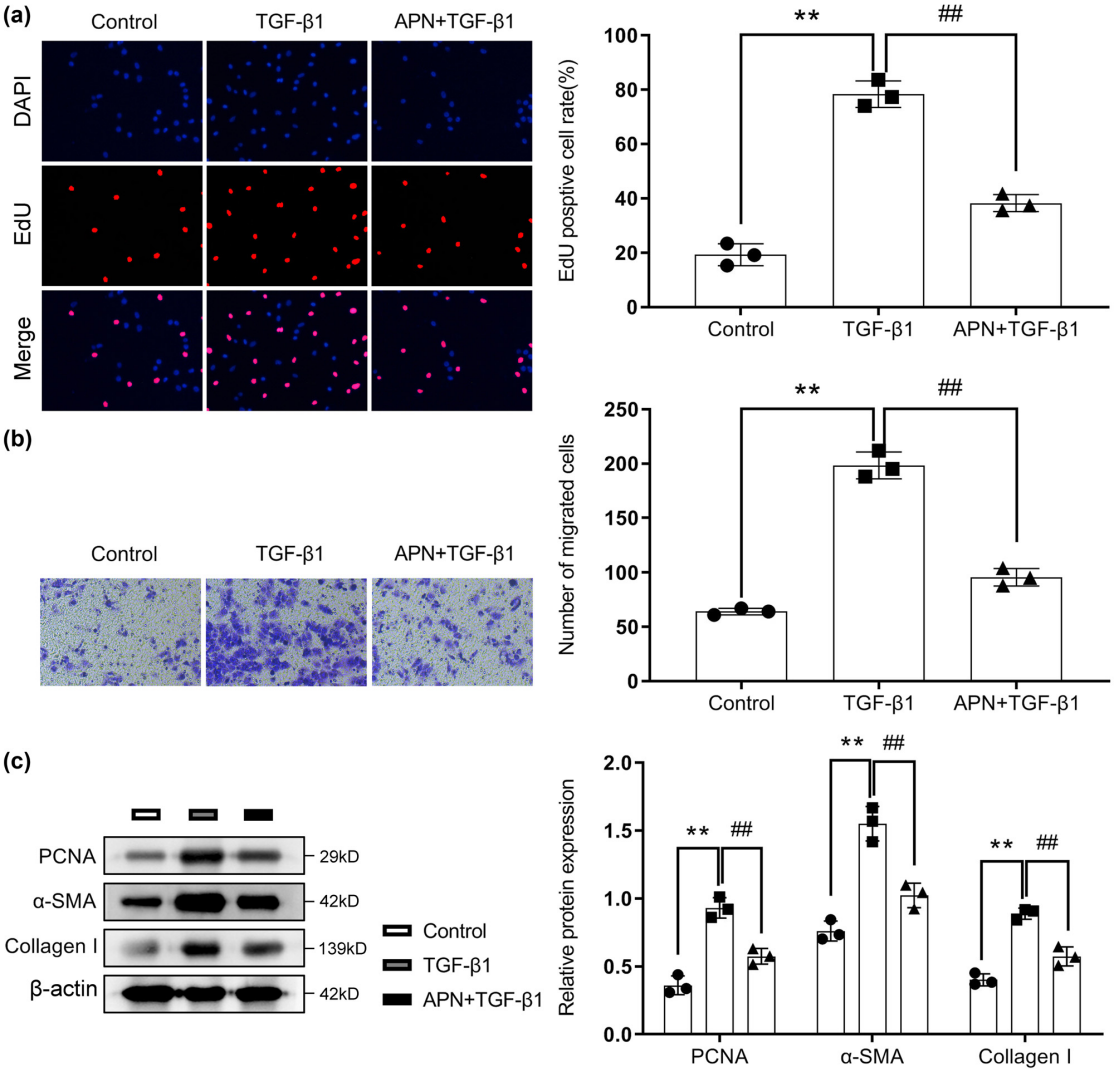
APN inhibits the phenotypic transformation of HSF induced by TGF-β1. The cells were treated with TGF-β1 (10 ng/mL) and APN (10 μg/mL). (a) Cell proliferation was detected by EdU assay. (b) Cell migration was detected by transwell assay. (c) The protein levels of PCNA, α-SMA and Collagen Ⅰ were detected by Western blot assay. Values are presented as the mean value ± SD (*n* = 3). **p < 0.01 compared to the control group. ##*p* < 0.01 compared to the TGF-β1 group.

**Figure 3:**
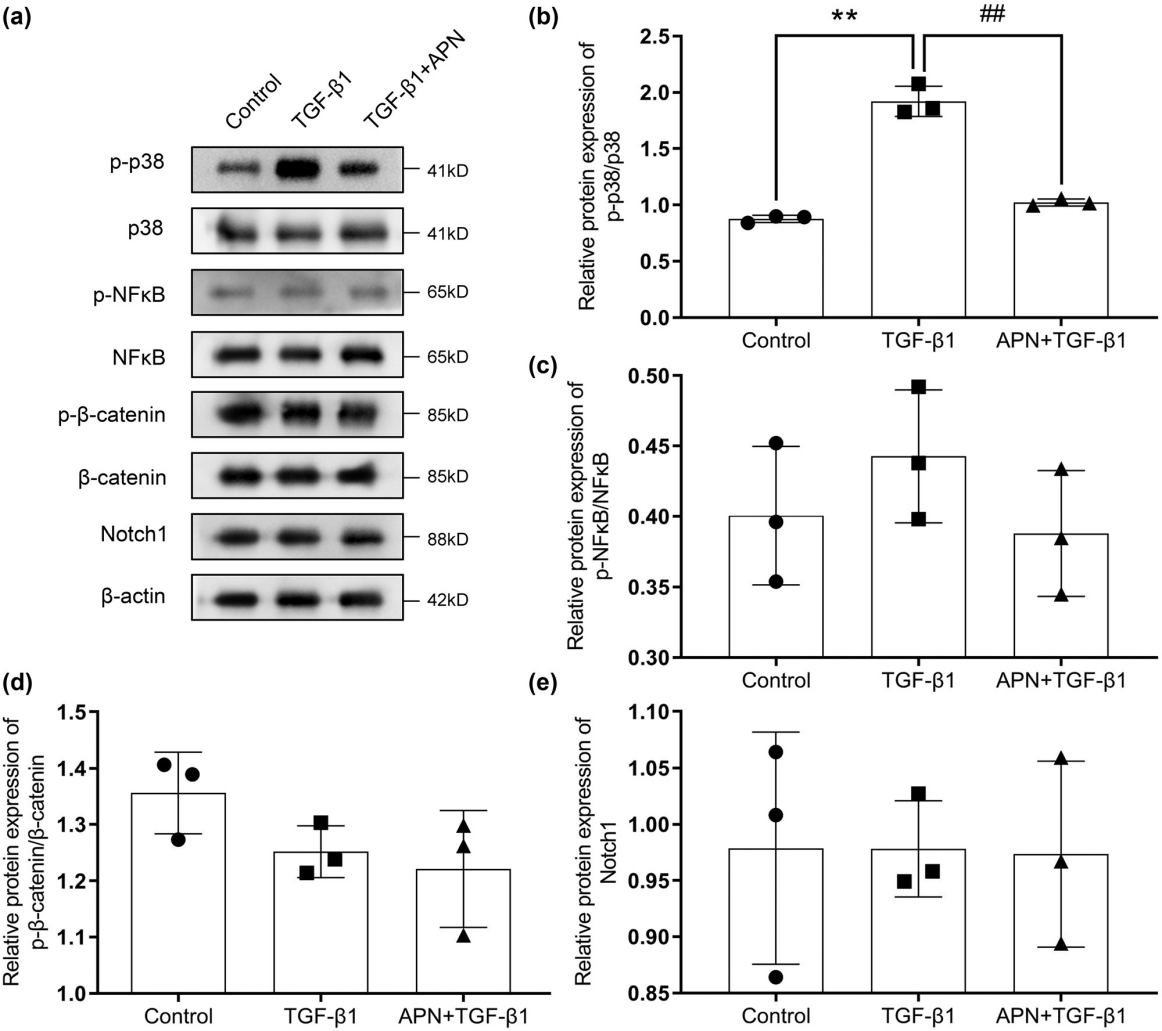
APN inhibits the p38 MAPK signaling pathway. The cells were treated with TGF-β1 (10 ng/mL) and APN (10 μg/mL). (a) The protein levels of the p38 MAPK, NF-κB, Wnt/β-catenin, and Notch1 signaling pathways were detected by Western blot. Quantification of the protein expression levels of p-p38 (b), p-NF-κB (c), p-β-catenin (d), and Notch1 (e). Values are presented as the mean value ± SD (*n* = 3). **p < 0.01 compared to the control group. ##*p* < 0.01 compared to the TGF-β1 group.

EdU and transwell assays were performed to explore the effects of APN on cell proliferation and migration in TGF-β1-treated HSF cells. We found that compared with the control group, the cell proliferation and migration of the HSF cells were significantly increased after TGF-β1 treatment, while APN treatment significantly decreased the cell proliferation and migration abilities of the TGF-β1-treated HSF cells (Figure 2a and b). Additionally, the total protein in the cells was extracted, and the protein expressions of PCNA, α-SMA, and collagen I were detected by Western blotting. As shown in Figure 3c, the protein expression levels of PCNA, α-SMA, and collagen I in the TGF-beta1 group were significantly higher than those in the control group, and APN treatment significantly increased the protein expression levels of PCNA, α-SMA, and collagen I in TGF-β1-treated HSF cells.

### 3.3 APN inhibits the p38 MAPK signaling pathway

Subsequently, we analyzed the effects of APN on cell growth-related pathways in TGF-β1-treated HSF cells (p38 MAPK, NF-κB, Wnt/β-catenin, and Notch1 signaling pathways). The results showed that TGF-β1 treatment significantly upregulated the p-p38 protein levels (Figure 3a and b) and showed no effects on the NF-κB (Figure 3a and c), Wnt/β-catenin (Figure 3a and d), and Notch1 (Figure 3a and e) signaling pathways. Additionally, after APN treatment, the p-p38 protein levels were significantly decreased in the TGF-β1-treated HSF cells and showed no effects on the NF-κB and Wnt/β-catenin signaling pathways (Figure 3a–e).

### 3.4 Blockade of p38 MAPK activity reversed TGF-β1-induced injury in HSF cells

**Figure 4:**
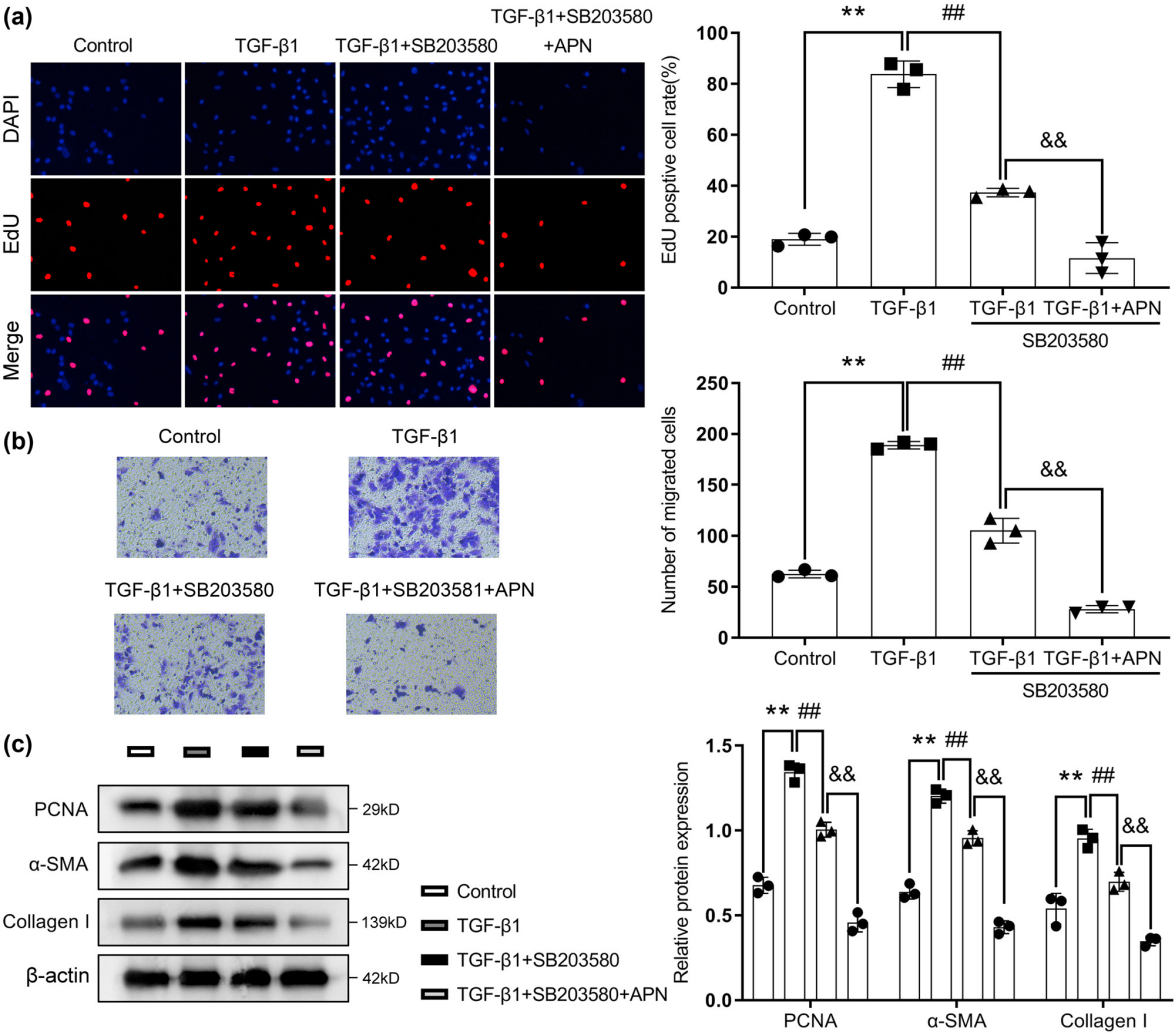
Blockade of p38 MAPK activity reversed TGF-β1-induced injury in HSF cells. The cells were treated with SB203580 (a p38 MAPK signaling pathway inhibitor), TGF-β1 (10 ng/mL), and APN (10 μg/mL). (a) Cell proliferation was detected by EdU assay. (b) Cell migration was detected by transwell assay. (c) The protein levels of PCNA, α-SMA, and collagen Ⅰ were detected by Western blot assay. Values are presented as the mean value ± SD (*n* = 3). **p < 0.01 compared to the control group. ##*p* < 0.01 compared to the TGF-β1 group. &&*p* < 0.01 compared to the TGF-β1 + APN group.

Finally, the cells were treated with SB203580 (a p38 MAPK signaling pathway inhibitor) for 1 h and then costimulated with TGF-β1 and APN. The results showed that SB203580 treatment relieved the effects of TGF-β1 on cell proliferation (Figure 4a) and migration (Figure 4b) in HSF cells. In addition, the upregulation of PCNA, α-SMA, and collagen I protein levels in the TGF-β1-treated HSF cells was significantly downregulated after SB203580 treatment (Figure 4c). Interestingly, SB203580 treatment promoted the therapeutic effects of APN on cell proliferation, migration, PCNA, α-SMA, and collagen I protein levels in TGF-β1-treated HSF cells (Figure 4a–c).

## 4 Discussion

Pathological scars are the inevitable result of abnormal wound healing after skin burn, trauma, and operation. The process by which pathological scars form is extremely complicated, and the current molecular mechanism research is still insufficient. Local medications, surgical treatment, laser therapy, and radiation are now the most popular forms of treatment, although no single approach has been proven to be completely effective. Clinicians and researchers are now focused on learning more about the pathophysiology of pathological scars and discovering efficient therapeutic options.

After skin injury, Fbs around the wound proliferate, migrate, and promote wound healing and tissue repair by synthesizing and secreting ECM, which is mainly composed of I/III collagen. The proliferation of Fb and the transition to the MFb phenotype are crucial for the functions of Fb [18]. The primary marker of Fb phenotypic transformation is the cytoplasmic expression of α-smooth muscle actin (α-SMA). With wound healing, the apoptosis of MFb in granulation tissue gradually disappeared [19]. In contrast to a healthy scar, there is a relative increase in the expression of cytokines that induce Fb proliferation and the deposition of ECM, while there is a relative decrease in the expression of proteins that promote apoptosis. Therefore, Fb is continuously transformed into the MFb phenotype, and MFb is constantly activated, leading to enhanced ECM synthesis and accumulation and formation of abnormally enlarged scars [3,5,20]. Thus, a pathological wound-healing process can lead to disorderly scar formation, which is characterized by excessive fibrosis. Exploring the fibrosis process of Fb proliferation and MFb phenotypic transition is therefore crucial for understanding the mechanism of scar formation and for successfully treating and preventing scarring.

TGF-β is believed to be a critical factor in regulating scar production and fibrosis caused by MFb activation [21]. The most active of these is TGF-β 1, which has the ability to interact directly with Fbs, encourage their migration and proliferation, and play a crucial role in the phenotypic transformation of skin Fbs by upregulating the expression of α-SMA and the synthesis of type I and type III collagen [22,23]. To mimic the development of pathological scars, HSFs were continually exposed to TGF-β1 in this experiment. Here we found that the proliferation, migration, and PCNA protein expressions of HSF were increased significantly after TGF-β1 induction, and the transformation to MFb was increased, suggesting that TGF-β1 inhibited the overexpression of ECM in scar tissue, which was consistent with the results of Bai et al. [24]. According to Meng et al., during wound healing, TGF-β1 secreted by various immune cells (such as macrophages and lymphocytes) synergizes with TGF-βRI on MFbs to activate the downstream Smad signaling pathway, and activated Smad 2/3 combined with Smad 4 enters the nucleus to regulate the expression of α-SMA, type I collagen, and matrix metalloenzyme tissue inhibitor, thus promoting the activation of MFbs and increasing the mechanical stress in scar tissue. TGF-β1 can also activate other non-Smad-dependent pathways (such as the MAPK, PI3K, and Rho-coupled GTPase signaling pathways) to promote the activation of MFbs. Therefore, blocking the expression of TGF-β1 or inhibiting its signaling pathway may be an effective method to prevent and treat pathological scars.

The mechanism and role of the TGF-β1/Smad classical signaling pathway in fibrotic diseases and pathological scar formation have been widely confirmed [25–28]. This study focuses on the p38 MAPK signaling pathway, which is a non-Smad-dependent signaling pathway. P38 MAPK is a tyrosine phosphorylated protein kinase, which is the cross point of the cell signaling pathway. To date, *in vivo* and *in vitro* experiments have confirmed that inhibiting the p38 MAPK signaling pathway can reduce scar formation and the expression of related factors [29,30]. *In vitro* studies have found that stem cells from adipose tissue reduce the expression of collagen I, collagen III, and α-SMA by inhibiting the p38 MAPK pathway [29]. Recent studies have shown that the expression of related factors of the p38 MAPK pathway in keloid tissues is significantly higher than that in normal skin and physiological scars [31]. In this study, we demonstrated that p38 kinase was highly phosphorylated following TGF-β1 stimulation. SB203580, an inhibitor of p38, can significantly reduce the expression of α-SMA induced by TGF-β1, indicating that TGF-β1 may promote the proliferation, migration, and phenotypic transformation of HSF through the p38 MAPK pathway.

Previous investigations have shown that APN, as a negative regulatory factor, plays an essential role in the pathogenesis of fibrotic and keloid diseases [32]. We performed western blot analysis of HSF to determine whether APN plays an antifibrotic role by inhibiting the p38 signaling pathway mediated by TGF-β1. The results showed that APN inhibited the proliferation and migration of HSF induced by TGF-β1. In addition, we found that APN inhibited the phenotypic transformation of HSF into MFbs induced by TGF-β1. Mechanistically, APN treatment inhibited the phosphorylation of p38 kinase induced by TGF-β1 in HSF. As reported by Lakota et al. [33], APN levels reflected PPAR-γ activity, and were correlated with skin fibrosis. APN might have potential utility as a biomarker in systemic sclerosis. Arakawa et al. [34] found that there was a significant reduction in serum APN levels in systemic sclerosis patients, which also indicated that serum APN levels may be a useful biomarker for fibrotic condition in systemic sclerosis patients with SSc. Compared with these research works, our study further delved into the regulatory mechanism of APN in skin fibrosis and pathological scars. Also, these results suggested targeting APN might be an effective method for skin fibrosis and pathological scars.

## 5 Conclusion

These studies indicated that APN might inhibit the proliferation, migration, and phenotypic transformation of HSF induced by TGF-β1 by inhibiting the p38 MAPK signaling pathway, which further confirmed the molecular mechanism by which APN inhibits HSF fibrosis at the cellular level. Therefore, APN is expected to become a potential target for the prevention and treatment of skin fibrosis and pathological scars. However, our study only discussed the *in vitro* mechanism by which APN inhibits skin fibrosis. Additional research on clinical patients and *in vivo* animal models is anticipated to corroborate these findings.

## References

[1] Karppinen SM, Heljasvaara R, Gullberg D, Tasanen K, Pihlajaniemi T. Toward understanding scarless skin wound healing and pathological scarring. F1000Res. 2019;8. 10.12688/f1000research.18293.1 PMC6556993 31231509

[2] Huang F, Shi ZJ, Lei Y, Yang QY. Effects of tripterine on viability and apoptosis of keloid fibroblasts by regulating GINS2. Chin J Pathophysiol. 2020;36:126–32.

[3] Schuster R, Younesi F, Ezzo M, Hinz B. The role of myofibroblasts in physiological and pathological tissue repair. Cold Spring Harb Perspect Biol. 2023;15(1):a041231. 10.1101/cshperspect.a041231 PMC9808581 36123034

[4] Garrison AT, Bignold RE, Wu X, Johnson JR. Pericytes: The lung-forgotten cell type. Front Physiol. 2023;14:1150028. 10.3389/fphys.2023.1150028 PMC10076600 37035669

[5] Schumacher D, Curaj A, Staudt M, Simsekyilmaz S, Kanzler I, Boor P, et al. Endogenous modulation of extracellular matrix collagen during scar formation after myocardial infarction. Int J Mol Sci. 2022;23(23):14571. 10.3390/ijms232314571 PMC9741070 36498897

[6] Wei Q, Liu D, Chu G, Yu Q, Liu Z, Li J, et al. TGF-β1-supplemented decellularized annulus fibrosus matrix hydrogels promote annulus fibrosus repair. Bioact Mater. 2022;19:581–93. 10.1016/j.bioactmat.2022.04.025 PMC9108517 35600980

[7] Oh J, Lee Y, Oh SW, Li T, Shin J, Park SH, et al. The role of adiponectin in the skin. Biomol Ther (Seoul). 2022;30(3):221–31. 10.4062/biomolther.2021.089 PMC9047493 34615771

[8] Nguyen TMD. Adiponectin: Role in physiology and pathophysiology. Int J Prev Med. 2020;11:136. 10.4103/ijpvm.IJPVM_193_20 PMC7554603 33088464

[9] Wang X, Yang J, Wu L, Tong C, Zhu Y, Cai W, et al. Adiponectin inhibits the activation of lung fibroblasts and pulmonary fibrosis by regulating the nuclear factor kappa B (NF-κB) pathway. Bioengineered. 2022;13(4):10098–110. 10.1080/21655979.2022.2063652 PMC9162013 35435119

[10] Čustović N, Rašić S. Relationship of serum adiponectin and resistin levels with the severity of liver fibrosis in patients with chronic hepatitis B. J Med Biochem. 2022;41(2):176–83. 10.5937/jomb0-33793 PMC9010048 35510200

[11] Zhao D, Zhu X, Jiang L, Huang X, Zhang Y, Wei X, et al. Advances in understanding the role of adiponectin in renal fibrosis. Nephrology (Carlton). 2021;26(2):197–203. 10.1111/nep.13808 33073881

[12] Meng K, Cai H, Cai S, Hong Y, Zhang X. Adiponectin modified BMSCs alleviate heart fibrosis via inhibition TGF-beta1/smad in diabetic rats. Front Cell Dev Biol. 2021;9:644160. 10.3389/fcell.2021.644160 PMC8019808 33829019

[13] Luo L, Li J, Liu H, Jian X, Zou Q, Zhao Q, et al. Adiponectin is involved in connective tissue growth factor-induced proliferation, migration and overproduction of the extracellular matrix in keloid fibroblasts. Int J Mol Sci. 2017;18(5):1044. 10.3390/ijms18051044 PMC5454956 28498357

[14] Luo L, Li J, Wu Y, Qiao J, Fang H. Adiponectin, but not TGF-β1, CTGF, IL-6 or TNF-α, may be a potential anti-inflammation and anti-fibrosis factor in keloid. J Inflamm Res. 2021;14:907–16. 10.2147/JIR.S301971 PMC7981148 33758530

[15] Darmawan CC, Montenegro SE, Jo G, Kusumaningrum N, Lee SH, Chung JH, et al. Adiponectin-based peptide (ADP355) inhibits transforming growth factor-β1-induced fibrosis in keloids. Int J Mol Sci. 2020;21(8):2833. 10.3390/ijms21082833 PMC7215791 32325772

[16] Zhang J, Li Y, Liu J, Han F, Shi J, Wu G, et al. Adiponectin ameliorates hypertrophic scar by inhibiting Yes-associated protein transcription through SIRT1-mediated deacetylation of C/EBPβ and histone H3. iScience. 2022;25(10):105236. 10.1016/j.isci.2022.105236 PMC9579505 36274941

[17] Guo Y, Chu G, Cai W, Li Y, Lan X, Li J, et al. Beneficial effects of oleosomes fused with human fibroblast growth factor 1 on wound healing via the promotion of angiogenesis. Int J Mol Sci. 2022;23(21):13152. 10.3390/ijms232113152 PMC9656666 36361940

[18] Xu R, Wu M, Wang Y, Li C, Zeng L, Wang Y, et al. Mesenchymal stem cells reversibly de-differentiate myofibroblasts to fibroblast-like cells by inhibiting the TGF-β-SMAD2/3 pathway. Mol Med. 2023;29(1):59. 10.1186/s10020-023-00630-9 PMC10131436 37098464

[19] Ortiz-Zapater E, Signes-Costa J, Montero P, Roger I. Lung Fibrosis and Fibrosis in the Lungs: Is It All about Myofibroblasts? Biomedicines. 2022;10(6):1423. 10.3390/biomedicines10061423 PMC9220162 35740444

[20] Song J, Gao H, Zhang H, George OJ, Hillman AS, Fox JM, et al. Matrix adhesiveness regulates myofibroblast differentiation from vocal fold fibroblasts in a bio-orthogonally cross-linked hydrogel. ACS Appl Mater Interfaces. 2022;14(46):51669–82. 10.1021/acsami.2c13852 PMC10350853 36367478

[21] Lodyga M, Hinz B. TGF-beta1 - A truly transforming growth factor in fibrosis and immunity. Semin Cell Dev Biol. 2020;101:123–39. 10.1016/j.semcdb.2019.12.010 31879265

[22] Liu J, Chen T, Zhao Y, Ding Z, Ge W, Zhang J. Blood donation improves skin aging through the reduction of iron deposits and the increase of TGF-β1 in elderly skin. Mech Ageing Dev. 2022;205:111687. 10.1016/j.mad.2022.111687 35697258

[23] Qin H, Zhang L, Li M, Liu Y, Sun S, Nie W, et al. EGR1/NOX4 pathway regulates oxidative stress and further facilitates fibrosis progression in keloids responses to TGF-β1. J Dermatol Sci. 2022;108(3):138–45. 10.1016/j.jdermsci.2022.12.009 36608994

[24] Bai X, He T, Liu J, Wang Y, Fan L, Tao K, et al. Loureirin B inhibits fibroblast proliferation and extracellular matrix deposition in hypertrophic scar via TGF-beta/Smad pathway. Exp Dermatol. 2015;24(5):355–60. 10.1111/exd.12665 25683490

[25] Gwon MG, An HJ, Kim JY, Kim WH, Gu H, Kim HJ, et al. Anti-fibrotic effects of synthetic TGF-beta1 and Smad oligodeoxynucleotide on kidney fibrosis in vivo and in vitro through inhibition of both epithelial dedifferentiation and endothelial-mesenchymal transitions. FASEB J. 2020;34(1):333–49. 10.1096/fj.201901307RR 31914629

[26] Zhang T, Wang XF, Wang ZC, Lou D, Fang QQ, Hu YY, et al. Current potential therapeutic strategies targeting the TGF-beta/Smad signaling pathway to attenuate keloid and hypertrophic scar formation. Biomed Pharmacother. 2020;129:110287. 10.1016/j.biopha.2020.110287 32540643

[27] Li YH, Yang J, Zheng Z, Hu DH, Wang ZD. Botulinum toxin type A attenuates hypertrophic scar formation via the inhibition of TGF-beta1/Smad and ERK pathways. J Cosmet Dermatol. 2021;20(5):1374–80. 10.1111/jocd.13842 33185943

[28] Xu S, Dong W, Shi Y. LncRNA PICSAR binds to miR-485-5p and activates TGF-beta1/Smad to promote abnormal proliferation of hypertrophic scar fibroblasts (HSFs) and excessive deposition of extracellular matrix (ECM). Med Mol Morphol. 2021;54(4):337–45. 10.1007/s00795-021-00296-4 34255190

[29] Ding Q, Yue J, Xue LF, Xu YX, Xiao WL. Inhibition of p38 mitogen-activated protein kinases may attenuate scar proliferation after cleft lip surgery in rabbits via Smads signaling pathway. Eur J Med Res. 2022;27(1):126. 10.1186/s40001-022-00757-1 PMC9301840 35858881

[30] Chai CY, Song J, Tan Z, Tai IC, Zhang C, Sun S. Adipose tissue-derived stem cells inhibit hypertrophic scar (HS) fibrosis via p38/MAPK pathway. J Cell Biochem. 2019;120(3):4057–64. 10.1002/jcb.27689 30260015

[31] Zhang MZ, Dong XH, Zhang WC, Li M, Si LB, Liu YF, et al. A comparison of proliferation levels in normal skin, physiological scar and keloid tissue. J Plast Surg Hand Surg. 2021;1–7. 10.1080/2000656X.2021.2017294 34964674

[32] Aljafary MA, Al-Suhaimi EA. Adiponectin system (Rescue Hormone): The missing link between metabolic and cardiovascular diseases. Pharmaceutics. 2022;14(7):1430. 10.3390/pharmaceutics14071430 PMC9321059 35890325

[33] Lakota K, Wei J, Carns M, Hinchcliff M, Lee J, Whitfield ML, et al. Levels of adiponectin, a marker for PPAR-gamma activity, correlate with skin fibrosis in systemic sclerosis: Potential utility as biomarker? Arthritis Res Ther. 2012;14(3):R102. 10.1186/ar3827 PMC3446479 22548780

[34] Arakawa H, Jinnin M, Muchemwa FC, Makino T, Kajihara I, Makino K, et al. Adiponectin expression is decreased in the involved skin and sera of diffuse cutaneous scleroderma patients. Exp Dermatol. 2011;20(9):764–6. 10.1111/j.1600-0625.2011.01310.x 21615510

